# Defining the breakpoints of the v *ermilion white* ( *v ^w^* ) mutation, a deletion that removes *vermilion, gustatory receptor candidate 58,* and *norpA* .

**DOI:** 10.17912/micropub.biology.002073

**Published:** 2026-04-01

**Authors:** Josy Joseph, Douglas Rusch, Andrew Zelhof

**Affiliations:** 1 Biology, Indiana University, Bloomington, IN, US; 2 Center for Genomics and Bioinformatics, Indiana University, Bloomington, IN, US

## Abstract

A key mutation for generating transgenics in *Tribolium castaneum* is *vermilion ^white^* ( *v ^w^* ). *&nbsp;v ^w^* is a deletion that removes most of the *vermilion* locus, but the upstream breakpoint has not been mapped. Here we report that the second breakpoint is located upstream in the *Tribolium* homolog of *norpA.* The *v ^w^* deletion is 4434 bps. The deletion eliminates not only *vermilion* but also *gustatory receptor candidate 58* and *norpA* function. Therefore, the *v ^w^* mutation is a deficiency that affects three genetic loci. To acknowledge the disruption of multiple loci this genetic mutant will be known as Deficiency *vermilion ^white^* , Df ( *v ^w^* ).

**Figure 1. Genomic organization of the v w deletion:** A. Schematic of *Tribolium castaneum* genetic loci of three genes affected by *vermilion ^white^* ( *v ^w^* ) deletion. B. Coverage or depth of sequence alignment at genomic region specific to the *v ^w^* deletion. Thirty nucleotides flanking each side of the 5′ and 3′ deletion breakpoints are highlighted, with red lines indicating the exact deletion boundaries. C. The amino acid sequence of exon 9 of *norpA* and the new open reading frame amino acids of exon 9 in *v ^w^* . There is a stop codon after 15 amino acids. D. Comparison of protein structure of Phospholipase C between *norpA* and truncated *norpA* reveals the PH domain, Phosphoinositide phospholipase C beta1-4-like EF-hand domain and Phosphatidylinositol-specific phospholipase C domains remain intact but the C2 domain is truncated.

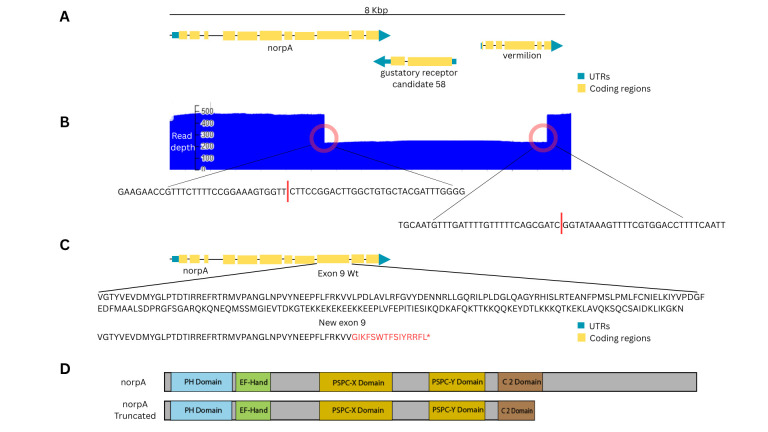



## Description

*Tribolium* is a widely utilized model organism for evolutionary and developmental biology questions and understanding regulatory mechanisms for agricultural pests (Adamski et al., 2019; Brown et al., 2009; Pointer et al., 2021; Rosner et al., 2020). *Tribolium* was the first Coleoptera genome to be sequenced (Tribolium Genome Sequencing Consortium, 2008), and an updated genome sequence based upon long read sequencing (icTriCast1.1 - (Childers et al., 2021) is now available. *Tribolium* is amenable to both forward and reverse genetics, and in particular transgenesis is well established (Campbell et al., 2022; Klingler & Bucher, 2022). For transgenics, the 3XP3 fluorescent marked transposable elements is an efficient marker (Berghammer et al., 1999; Horn et al., 2000) and easily detected in *Tribolium* mutant retinas that lack pigmentation, e.g. *pearl* , *platinum* and *white* . Lorenzen et al. demonstrated that the *Tribolium* *white* mutation (Eddleman & Bell, 1963) is a null mutation of the *Tribolium* homolog of *vermilion* (Lorenzen et al., 2002a). Moreover, given the absence of pigment in *vermilion* mutant retinas, a set of transposable elements have been generated that result in the expression of *vermilion* and thus restores pigmentation to the retina (Lorenzen et al., 2002b). As a result, many *Tribolium* transgenics are generated in the *v ^w^* mutant background.

&nbsp;

The initial characterization of *vermilion ^white^* demonstrated that the mutant is a deletion that removes the first five exons and extends into the last sixth exon of *vermilion* ( Figure 1A ). &nbsp;The upstream breakpoint was not mapped. To map the upstream breakpoint, we took advantage of PacBio long read sequence that contained the *v ^w^* mutation. Our sequencing revealed a 4,434 bp deletion. The sequence confirmed the break point that lies within *vermilion* and identified the upstream second breakpoint ( Figure 1B ). The deletion eliminates the entire *gustatory receptor candidate 58 locus* and extends into the *Tribolium* *norpA* locus. *norpA* encodes a Phospholipase C which is critical for phototransduction and in its absence vision is disrupted (Bloomquist et al., 1988). The deletion results in a 3’ prime deletion of exon 9 of the *norpA* locus resulting in the truncation of the C2 domain of the phospholipase and an addition of 15 unrelated amino acids Figure 1C, D). The C2 domain is 91&nbsp;amino-acid residues and thought to be involved in calcium-dependent phospholipid binding and membrane targeting (Davletov & Sudhof, 1993). The loss of the C2 domain can have multiple effects. It can prevent the protein from binding to the lipid bilayers (Croessmann et al., 2018) or it can impair the protein’s ability to respond to the calcium signals (Corbalan-Garcia & Gomez-Fernandez, 2014). The truncation can also reduce the stability of the protein, leading to premature degradation (Buetow & Huang, 2016). Thus the truncation of the C2 domain would suggest that the mutated *norpA* allele in the *v ^w^* mutation is a loss of function allele.

&nbsp;

With respect to the generation of transgenics, one will need to account for the additional mutations in *v ^w^* but this concern can be alleviated using CRISPR/Cas9 generated *vermilion* alleles (Adrianos et al., 2018; Markley et al., 2024). Whereas the existence of two additional mutations may decrease the usefulness of *v ^w^* with respect to transgenics, the existence of defined deletions has been critical for mapping of genes and confirming the nature of alleles. Moreover, the *v ^w^* mutation now adds a key mutation to the toolbox of understanding sensory perception. The uncovering of the *norpA* mutation can now help define the dynamics of phototransduction in *Tribolium* and permit an investigation in the role of vision in *Tribolium* behaviors, e.g. circadian rhythms. Overall due to the elimination of two other loci we propose that the *v ^w^* mutation is now referred as Deficiency *vermilion ^white^* , Df ( *v ^w^* ).

&nbsp;

&nbsp;

## Methods

***Tribolium* lines and husbandry:** All animals were raised at 28 ^o^ C on a standard flour yeast mix. The following strains were utilized: *vermilion ^white^* ( *v ^w^* ), and a strain heterozygous for *vermilion ^white^* ( *v ^w^* ) (Lorenzen et al., 2002a) and *Lucifer* ( *Lu* ) (Haas & Beeman, 2012).

**Sequencing and genome assembly:** 40 < 1 day old *vermilion ^white^* ( *v ^w^* )&nbsp; and *Lucifer* ( *Lu* ) heterozygote pupae were isolated and immediately frozen in liquid nitrogen and stored at –80 ^o^ C. Pupae were shipped on dry ice to Psomagen ( https://www.psomagen.com/ ) for processing. DNA extraction, library construction, and PacBio sequencing were all performed by Psomagen. The PacBio hifi reads were assembled with hifiasm (v0.25.0-r726) (Cheng et al., 2024; Cheng et al., 2021; Cheng et al., 2022). The resulting assemblies as well as the input reads were mapped to the reference genome *Tribolium castaneum* strain icTriCast1.1 using minimap2 (v2.28) (Li, 2018) with default parameters. This identified a 4434 bp deletion. Among reads that span the deleted region, we identified 139 reads without the deletion and 149 reads that contain the deletion. The sequencing reads have been deposited in the SRA database and have the following accession numbers: SRR37271882 : wild-type *vermilion* locus reads&nbsp;and SRR37271883 : *v ^w^* deletion locus reads.

***norpA* protein structure** : The protein domains were compiled using InterPro (Blum et al., 2025).
